# Nano-volcanic Eruption of Silver

**DOI:** 10.1038/srep34769

**Published:** 2016-10-05

**Authors:** Shih-kang Lin, Shijo Nagao, Emi Yokoi, Chulmin Oh, Hao Zhang, Yu-chen Liu, Shih-guei Lin, Katsuaki Suganuma

**Affiliations:** 1 Department of Materials Science and Engineering, National Cheng Kung University, Tainan, 70101, Taiwan; 2 Center for Micro/Nano Science and Technology, National Cheng Kung University, Tainan, 70101, Taiwan; 3 Institute of Scientific and Industrial Research, Osaka University, 8–1 Mihogaoka, Ibaraki, Osaka, Japan; 4 Department of Adaptive Machine Systems, Osaka University, 8–1 Mihogaoka, Ibaraki, Osaka, Japan

## Abstract

Silver (Ag) is one of the seven metals of antiquity and an important engineering material in the electronic, medical, and chemical industries because of its unique noble and catalytic properties. Ag thin films are extensively used in modern electronics primarily because of their oxidation-resistance. Here we report a novel phenomenon of Ag nano-volcanic eruption that is caused by interactions between Ag and oxygen (O). It involves grain boundary liquation, the ejection of transient Ag-O fluids through grain boundaries, and the decomposition of Ag-O fluids into O_2_ gas and suspended Ag and Ag_2_O clusters. Subsequent coating with re-deposited Ag-O and the de-alloying of O yield a conformal amorphous Ag coating. Patterned Ag hillock arrays and direct Ag-to-Ag bonding can be formed by the homogenous crystallization of amorphous coatings. The Ag “nano-volcanic eruption” mechanism is elaborated, shedding light on a new mechanism of hillock formation and new applications of amorphous Ag coatings.

Silver (Ag) is one of the seven metals that were used by ancient civilizations. Today, Ag is an important engineering material in the electronic, medical, and chemical industries and being a precious material for decorations, arts, and the storing of wealth. This wide range of applications of Ag is attributable to its unique noble and catalytic properties. In particular, the interactions of Ag with oxygen (O) are of great interests in various fields. The absorption and desorption of O onto and from Ag determine its heterogeneous catalytic capacity. Ag thin films have various microelectronic applications primarily owing to their oxidation-resistant property, consistent with the prediction of Ellingham^1^. Recently, several phenomena and modern applications of Ag have been reported, such as its liquid-like pseudoelasticity^2^, abundant hillock formation^3^, memristive switching^4^, low-temperature sintering^5^, and pressure-less direct bonding^6^. This study reports a newly identified phenomenon of “Ag nano-volcanic eruption” and elucidates its formation mechanism, which originates in, and is coupled with, Ag-O interactions. The mechanisms of abundant Ag hillock formation and pressure-less direct Ag-to-Ag bonding are explained.

## Results

### Formation of “Patterned” Abundant Ag Hillocks

**Figure 1:**
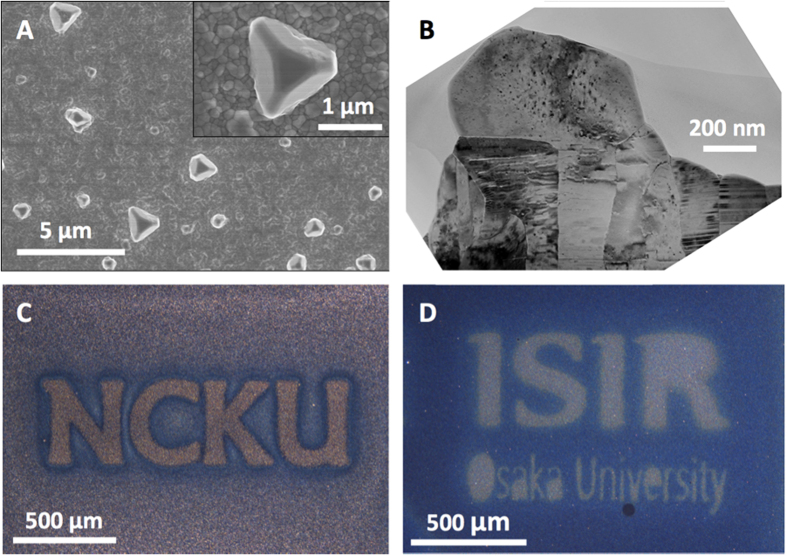
Abundant Ag hillocks formed on a sputtered Ag film, which was covered with a dummy Si chip and annealed at 250 °C under the ambient atmosphere for 1 h: (**A**) A top-view secondary electron image (SEI) of abundant Ag hillocks; and (**B**) The cross-sectional transmission electron microscope (TEM) micrograph of an individual abundant Ag hillock. Optical micrographs of patterned abundant Ag hillocks formed on a sputtered Ag film after being annealed at 250 °C for 1 h at (**C**) the “covered areas, *i.e*., inverse areas of NCKU” under the ambient atmosphere and (**D**) the “uncovered areas, *i.e*., ISIR/Osaka University” under an ultra-high vacuum condition (*P* < 10^−2^ Pa).

Ag is the first material for which metallic whisker formation was identified: the phenomenon was discovered as early as the 16th century^7^,^8^. The formation of whiskers involves growth of filament or nodule-type hillocks on the free surface. After the discovery of Ag whiskers, the formation of metallic whisker was also found in various metals, *e.g*., cadmium, zinc, tin, lead, and their alloys. The mechanism of whisker formation has been extensively studied over the past few decades, primarily due to the serious concerns for whisker-induced failures in electronic, military, and aerospace devices. The whisker formation is identified as a general phenomenon of stress relaxation and compressive stresses have been identified as the fundamental driving force for metallic hillock or whisker formation^9^. The “oxide-breaking” mechanism is generally accepted to be the mechanism of metallic whisker or hillock formation: most of the stressed atoms diffuse through grain boundaries, break through the oxide layer and accumulate locally at the free surface, forming whiskers or hillocks to release the stress^9^. However, it failed to explain the recently observed abundant Ag hillock formation on sputtered Ag films (Fig. 1A,B) at elevated temperatures^3^. Based on the standard Gibbs free energy of formation of silver oxide (Ag_2_O)^10^:


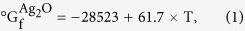


as given by the reaction:


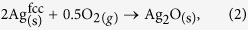


silver oxide (Ag_2_O) spontaneously decomposes to fcc-(Ag) and O_2(g)_ phases in air at 145 °C and higher temperatures. The above-mentioned “oxide-breaking” mechanism does not explain abundant Ag hillock formation, as the silver oxide on the free surface is not stable at the elevated temperatures.

The abundant Ag hillocks significantly roughened Ag films. Therefore, the formation of abundant Ag hillocks can be clearly identified using a conventional optical microscope (see supplementary materials). As presented in Fig. 1C,D, Ag hillocks are intriguingly only found in certain areas of Ag films under a specific “covering condition” with masks and corresponding atmosphere following heat treatments at an elevated temperature, such as 250 °C; these areas are either “covered areas (inverse parts of characters)” where the Ag film is gently covered by a patterned mask without intimate contact under the ambient atmosphere (P ~ 10^5^ Pa) (Fig. 1C), or “uncovered areas (characters)” under a high vacuum condition (P < 10^−2^ Pa) (Fig. 1D). To the best of the authors’ knowledge, this is the first report of “patterned” hillocks in the literature, as spontaneous whisker or hillock formation is known to be uncontrollable. The discovered patterned Ag hillocks may have biomedical, optoelectronics and catalytic applications, among others. However, no existing theory concerning whisker or hillock formation addresses the formation of abnormal patterned Ag hillocks.

### Pressure-less Direct Ag-to-Ag Bonding

**Figure 2:**
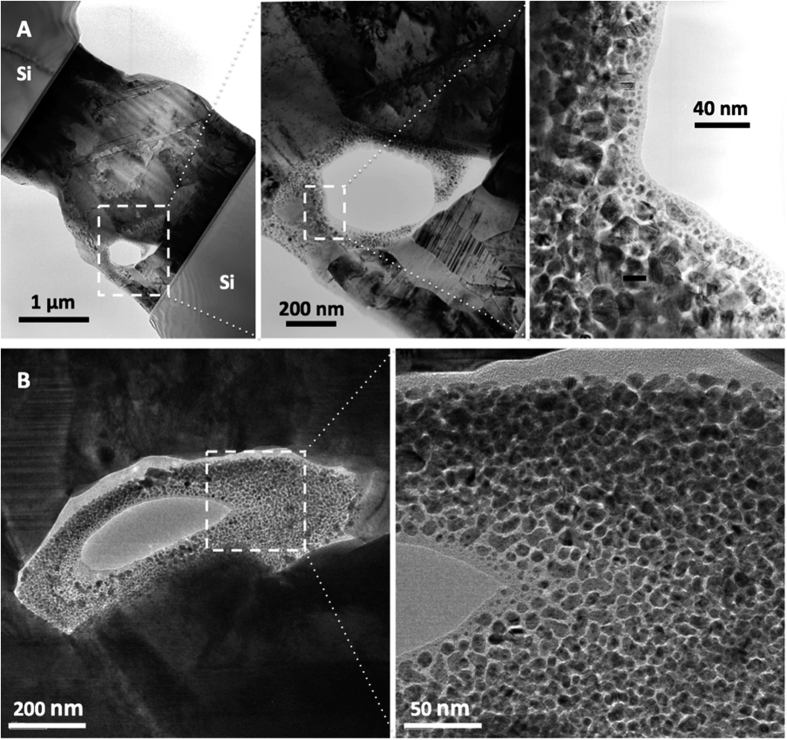
Cross-sectional TEM images of Ag/Ag joint bonded using 1 μm-thick sputtered Ag films at 250 °C under the ambient atmosphere for 1 h: (**A**) overview image and its close-ups of a joint and (**B**) overview image and its close-up of the other joint.

Pressure-less, low-temperature Ag-to-Ag bonding has recently been developed for die-attachment in advanced wide bandgap (WBG) electronics^6^,^11^,^12^. Compressive stress has been proposed to be the fundamental driving force of direct metal bonding in various systems, such as Ag-to-Ag, gold (Au)-to-Au, and copper (Cu)-to-Cu interconnections^6^,^11^,^12^,^13^,^14^,^15^. Unlike Cu-to-Cu bonding, which typically involves rather complex processes and strict processing environments^13^, Ag-to-Ag bonding, which exhibits extraordinary mechanical properties, can be established simply by employing sputtered Ag contacts^6^,^11^,^12^. The formation of strong Ag/Ag joints has also been considered to be a stress-driven phenomenon, which is associated with the aforementioned formation of abnormal abundant Ag hillocks^3^. However, as presented in Fig. 2, upon bonding at 250 °C under the ambient atmosphere for 1 h, nano-crystalline Ag grains are surprisingly dispersed in the amorphous Ag margins that are enclosed in large Ag grains at joints. The sizes of the nano-crystalline Ag grains range from ~2 to ~20 nm. The conventional stress migration mechanism cannot explain the formation of nano-crystalline Ag grains at Ag/Ag joints.

## Discussion

### Conventional Stress-migration Mechanism

The formation of abundant Ag hillocks and the pressure-less Ag-to-Ag bonding have both been attributed to the stress-migration mechanism that is driven by thermal stress and the residual stresses in sputtered Ag films^3^,^6^,^11^,^12^. The very large stress gradient has been estimated by X-ray diffractometry to be as high as 200 MPa^6^,^11^, and the mass transfer is associated with the columnar grain boundaries that are perpendicular to the substrate^3^. However, experiments have shown that the primary driving force for diffusion, which is the very large thermal-stress gradient, diminished instantly in the very early stage of annealing^11^. From the size and density of the abundant Ag hillocks, the mean volume of each Ag hillock is calculated based on stress migration to be ~0.2 μm^3^ (see kinetic analyses in supplementary materials), which is significantly smaller than that of abundant Ag hillocks (1~2 μm^3^) being observed in experiments^3^,^6^,^11^,^12^. The mechanism of stress migration along grain boundaries failed to quantitatively validate the mass-balance.

If Ag hillocks were formed by atomic diffusion or stress migration, then the energetically favorable mode would be either epitaxial growth of existing grains in the film, or nucleation as new grains at the junctions of grain boundaries close to the free surface, which are fast diffusion paths^16^,^17^. However, as presented in Fig. 1A,B, abundant Ag grains are surprisingly formed independently over a number of existing columnar grains in the film. The conventional compressive stress-induced diffusion (stress migration) mechanism cannot explain the grain morphology of the abundant Ag hillocks or the dependence of hillock formation on the “covering condition” and the corresponding atmosphere: Ag hillocks are formed in covered areas under the ambient atmosphere (Fig. 1C) and in uncovered areas under a high vacuum (Fig. 1D). Furthermore, the stress migration mechanism does not underlie the formation of nano-crystalline Ag grains or the amorphous Ag margin in Ag/Ag joints (Fig. 2).

The above controversial results suggest that the formation of abundant Ag hillocks and strong Ag/Ag joints involves a yet to be identified mechanism. Therefore, although the stress migration mechanism has been successfully used to explain the formation of metallic whiskers and pressure-assisted direct metal bonding in general, the mechanisms of formation of abundant Ag hillocks and Ag/Ag joints remain unclear, but they are associated with the following questions. (1) Why did independent crystals of abundant Ag hillock nucleate over a number of existing grains in the Ag film? (2) How did the extremely fast mass transfer proceed, forming abundant Ag hillocks and strong Ag/Ag joints? (3) Why did the formation of Ag hillocks depend on the “covering condition” in a given atmosphere? (4) Why did nano-crystalline Ag grains and amorphous Ag films form at the margin that was enclosed in large Ag grains in Ag/Ag joints?

### Nano-volcanic Eruption of Ag

**Figure 3:**
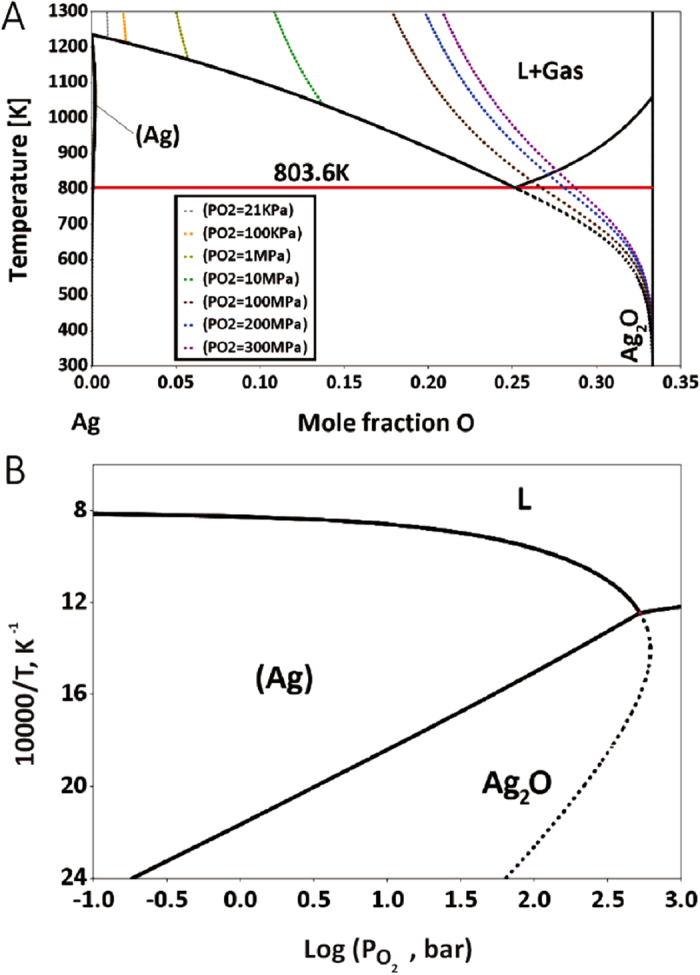
Calculated (**A**) phase diagram and (**B**) potential diagram of the Ag-O binary system with superimposed meta-stable equilibria between (Ag) and *L* phases.

To answer the above-mentioned questions, the phase equilibria of the Ag-O binary system are evaluated from fundamentals, based on the thermodynamic model proposed by Assal *et al*.^10^ (see the calculated phase diagram in Fig. 3A and the calculated potential diagram in Fig. 3B). The melting temperature (liquidus temperature) of the fcc-(Ag) phase declines as the partial pressure of oxygen increases. This phenomenon was firstly discovered experimentally in 1932^18^ and was more extensively studied in the 60 s^19^,^20^,^21^. Baker and Johnstone experimentally identified a clear reduction of liquidus temperatures as the partial pressure of oxygen in 1965^19^. When the pressure of oxygen reaches 530 atm (ca. 53.69 MPa), the melting temperature of Ag drops to 530 ± 4 °C^21^. Although the variations in lattice stability of fcc-Ag and Ag_2_O phases with pressure are negligible, with energy increases of less than 0.5 meV/atom at external pressures of up to 350 MPa (see *ab initio* calculations in supplementary materials), the interactions between Ag and O change the relative stability of the fcc and liquid phases in the Ag-O binary system. Based on the thermodynamic prediction in Fig. 3, a eutectic reaction:


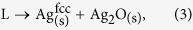


proceeds at 531 °C when the partial pressure of oxygen exceeds 52.6 MPa^10^. However, if the Ag_2_O phase could not nucleate, then the meta-stable melting points of fcc-(Ag), which are the meta-stable equilibria between the fcc-(Ag) and liquid phases, would fall drastically to low temperatures, as indicated in the dashed lines that are superimposed on Fig. 3A,B. Although the phase stability under extremely high partial pressures of oxygen without Ag_2_O nucleation has not been verified owing to experimental difficulties, this trend of liquation and extrapolation of stable to meta-stable liquidus have been clearly interpreted using thermodynamic modeling based on available experimental information.

Oxygen atoms are well known to penetrate Ag films readily trough grain boundaries. Therefore, O atoms are fully absorbed at Ag grain boundaries and are in equilibrium with the ambient oxygen gas prior to heat treatment. These absorbed O and Ag atoms are not likely to nucleate into Ag_2_O phase because they are constrained at grain boundaries. Rather, oxide-like structures may be present with buckled O atoms beneath the superficial Ag atoms of each grain^22^. The buckling-structured Ag-O mixtures at grain boundaries are with a low melting temperature at high partial pressures of oxygen. During heat treatment, a very large thermal-stress of the order of hundreds of MPa is generated owing to the large mismatch of coefficients of thermal expansion (CTE) between Ag and the Si substrate^6^. This great stress during heat treatment of the Ag-O mixture induces grain boundary liquation. The critical temperature of Ag-O grain boundary liquation is determined to be approximately 150 °C, which is close to the decomposition temperature of Ag_2_O (see supplementary materials). Meanwhile, the Ag-O fluids at the grain boundaries are squeezed out toward the free surface, so the large compressive stresses are relieved very quickly in the early stage of the reaction and the extremely fast mass transfer of Ag occurs.

Following the sudden reduction of pressure and temperature by ejection, the Ag-O fluids are no longer stable and instantly undergo the solidification reaction:


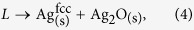


such that the ejected Ag-O fluids are transformed into suspended Ag and Ag_2_O clusters. If the temperature of the suspended Ag_2_O clusters still exceeds the normal decomposition temperature (145 °C), then the decomposition reaction:


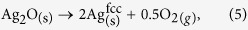


simultaneously occurs; if the suspended Ag_2_O clusters are quenched to a lower temperature after ejection, then the oxide form is retained. Therefore, the overall reaction is


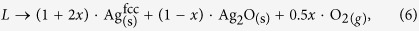


where *x* is the number of moles of decomposed Ag_2_O_(s)_ per mole of A-O fluid (*L*). The Ag-O fluids that are ejected from grain boundaries are transformed into O_2_ gas and free Ag and Ag_2_O clusters, exactly as in a volcanic eruption. The suspended Ag and Ag_2_O clusters are then re-deposited on the Ag film, just as ash falls after a volcanic eruption, forming a conformal Ag-O coating. Since the temperature of the Ag film is at 250 °C, above the normal decomposition temperature of Ag_2_O, oxygen atoms of the Ag-O coating are de-alloyed and escape as O_2_ gas, leaving a conformal amorphous Ag coating behind.

**Figure 4:**
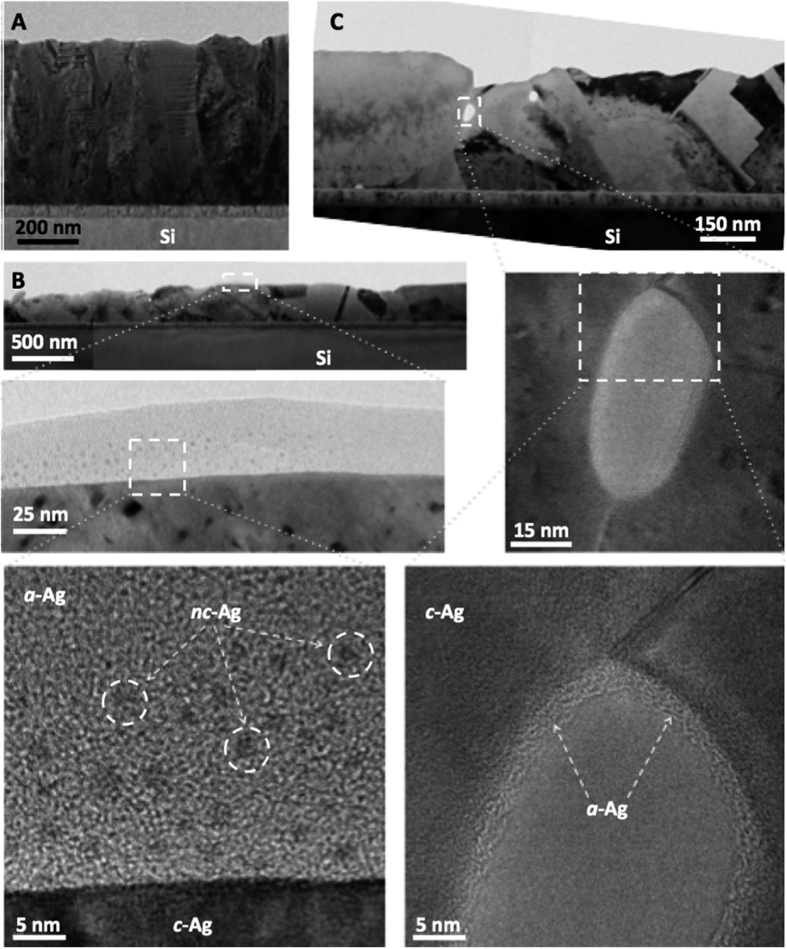
Cross-sectional TEM images of sputtered Ag films: (**A**) as-sputtered, and (**B,C**) annealed at 250 °C for 5 min, where *a*-Ag, *c*-Ag, and *nc*-Ag stand for amorphous Ag, crystalline Ag, and nano-crystalline Ag, respectively.

Direct evidence of the formation of amorphous Ag coating on sputtered Ag films can be observed in early stage of annealing. Figure 4A shows cross-sectional TEM images of an as-sputtered Ag film, and Fig. 4B,C show overview and magnified TEM images of two independent 5 min.-annealed Ag films, respectively. Columnar grains are observed in both as-sputtered and gently annealed Ag films. However, the sharp boundary of the free surface is seen only in the as-sputtered crystalline Ag (*c*-Ag) film (Fig. 4A), while both 5 min.-annealed Ag films are coated with conformal amorphous Ag (*a*-Ag) layers (Fig. 4B,C). As shown in the close-ups of *a*-Ag layer (Fig. 4B), the a-Ag layer is as thick as tens of nm with dispersive nano-crystalline Ag (*nc*-Ag) grains with diameters of ~1 nm. In addition to the free surface of Ag film (Fig. 4B), *a*-Ag coating was also found at interior surface of pores of Ag film (Fig. 4C). This finding strongly supports the “nano-volcanic eruption” mechanism of the formation of amorphous Ag coating on sputtered Ag films. Nano-crystalline Ag grains are formed through homogeneous nucleation within the amorphous Ag coating.

The formation of abundant Ag hillocks can be understood as the homogeneous crystallization of an amorphous Ag coating and the subsequent Ostwald ripening of nano-crystalline grains, explaining why the Ag hillocks formed herein as independent abundant crystals over a number of existing columnar grains in the Ag film (Fig. 1A,B). This “nano-volcanic eruption” mechanism also explains why nano-crystalline Ag grains and amorphous Ag margins formed in enclosed areas of large Ag grains in Ag/Ag joints (Fig. 2). Evidently, the rapid formation of a strong Ag-Ag joint is driven by the crystallization of the interfacial amorphous Ag layers, whereas the local equilibrium of the Ag-O coating and O_2(g)_ is established in enclosed areas. Thus, these peculiar phenomena of abundant Ag hillock formation and pressure-less Ag-to-Ag direct bonding both originate from the amorphous Ag coating. The universal “Ag nano-volcanic eruption” mechanism not only is a newly identified mechanism of hillock formation, but also provides a new method for forming amorphous Ag coatings, opening the door to new applications that involve amorphous Ag coatings, such as direct metal bonding.

Given a full understanding of the “nano-volcanic eruption” mechanism of Ag hillock formation, the formation of patterned Ag hillocks formed in “covered areas” under the ambient atmosphere (Fig. 1C) and “uncovered areas” in a vacuum (Fig. 1D) would not be surprising. Since Ag hillocks are nucleated from the amorphous Ag coating and the amorphous Ag coating is formed by the re-deposition of suspended Ag and Ag_2_O clusters and the subsequent de-alloying of O, the airflow near the Ag film critically determines the distribution of the Ag-O re-deposits and thus the amorphous Ag layers. Under the ambient atmosphere, as shown in Fig. 1C, the thermal convection-induced airflow may “blow” the suspended Ag away, while the mask acts as a “tent” that keeps the suspended Ag and Ag_2_O clusters in the “covered areas”. Hence, abundant Ag hillocks form only in the “covered areas” under the ambient atmosphere. However, no airflows under a vacuum: only the produced O_2_ gas flows, so the suspended Ag and Ag_2_O clusters are re-deposited at the original positions. However, the production of O_2_ gas in “covered areas” generates a radial pressure gradient from the center of the mask toward the uncovered areas, causing the suspended Ag and Ag_2_O clusters to flow out and be deposited in the “uncovered areas”, leaving an uncoated Ag surface behind in “covered areas” as shown in Fig. 1D.

**Figure 5:**
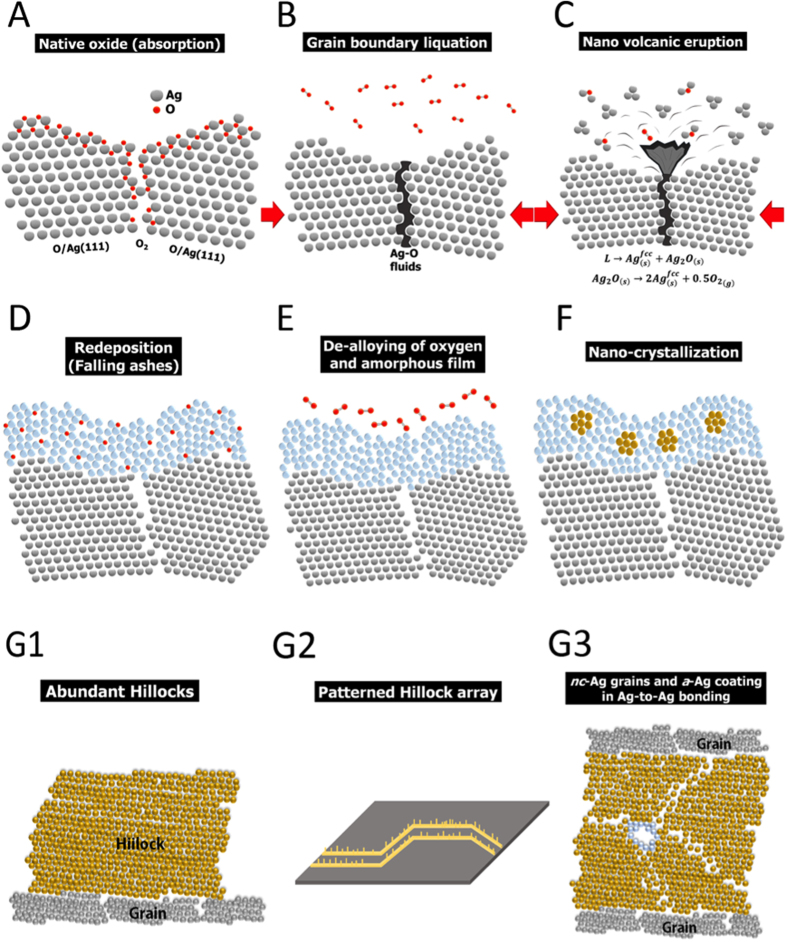
Schematic diagram of Ag nano-volcanic eruption: (**A**) Native oxide, (**B**) grain boundary liquation, (**C**) nano-volcanic eruption of Ag and Ag_2_O clusters and O_2_ gas, (**D**) re-deposition of Ag-O coating (falling ashes), (**E**) de-alloying of O and formation of amorphous Ag film, (**F**) homogeneous nucleation of nano-crystalline Ag grains, and formation of (G1) abundant Ag hillocks, (G2) patterned Ag hillock arrays, or (G3) strong Ag/Ag joints.

Figure 5 schematically depicts the Ag “nano-volcanic eruption”-assisted formation of an amorphous Ag film, an abundant Ag hillock, a patterned Ag hillock array, and an Ag/Ag joint. At room temperature, before heat treatment, the free surface and the grain boundaries of the Ag film fully absorb oxygen (Fig. 5A). Upon heat treatment, the absorbed oxygen at the surface desorbs, leaving pure Ag behind, while several instantaneous reactions occur simultaneously at grain boundaries; these include grain boundary liquation (Fig. 5B), ejection of the Ag-O fluid, quenching of the Ag-O fluid and partial decomposition of the solidified Ag_2_O (Fig. 5C), re-deposited free Ag and Ag_2_O clusters (Fig. 5D), and de-alloying of O from the re-deposited Ag-O coating (Fig. 5E). Accordingly, a conformal amorphous Ag coating is formed. During the subsequent heat treatment, homogeneous nucleation of the amorphous coating occurs (Fig. 5F), forming abundant Ag hillocks (Fig. 5G1), patterned Ag hillock arrays (Fig. 5G2), or strong Ag/Ag joints (Fig. 5G3).

This paper reports on a newly identified phenomenon of Ag nano-volcanic eruption, which involves several transient interactions between Ag and O, which can be exactly analogized to a volcanic eruption and the deposition of ash. An amorphous pure metal film/coating is very unstable and so very difficult to fabricate. The nano-volcanic eruption and subsequent de-alloying mechanisms can be used easily to grow a conformal amorphous Ag coating. Following homogeneous nucleation and the grain growth of grains in amorphous Ag coatings can be carried out to form abundant Ag hillocks and to induce pressure-less Ag-to-Ag direct bonding. Moreover, the fact that this amorphous coating can be controlled by the controlling the covering condition using masks and varying the processing atmosphere is exploited herein to demonstrate uniformly patterned Ag hillock arrays herein for the first time, opening the door to new applications of one of the most important metals in the history of civilization, silver.

## Materials and Method

### Materials and Fabrication

Three types of samples were fabricated in this study; they are abundant Ag hillocks, patterned Ag hillock arrays, and directed Ag-to-Ag bonded (Ag/Ag) joints. For all samples, 1.0 μm-thick Ag films were sputtered on 0.04 μm-thick Ti-coated Si substrates. The Si substrates were prepared using a 500 μm-thick Si wafer, which was cut into pieces with the dimension of 6 mm x 6 mm and ultrasonically cleaned with acetone for 5 min prior to sputtering. The Ti adhesion layers and Ag films were subsequently deposited using radio frequency (RF) sputtering and direct current (DC) sputtering, respectively. The base pressure of the sputtering vacuum chamber was lower than 5.0 × 10^−3^ Pa, the Ar flow rate was 10 sccm (standard cubic centimeters per minute), and the substrate temperature for the deposition process was at 25 °C. The growth rate of Ti and Ag films was 6 nm/min and 30 nm/min, respectively. For fabricating abundant Ag hillocks, patterned Ag hillock arrays, or Ag/Ag joints, dummy chips (without metallization), masks, or upside-down metalized (Si/Ti/Ag) dummy chips were placed on the Ag/Ti/Si substrates, respectively. The dummy chips were also prepared using a 500 μm-thick Si wafer, which was cut into pieces with the dimension of 3 mm × 3 mm, ultrasonically cleaned with acetone for 5 min, and treated with or without sputtering. The masks were fabricated from 30 μm-thick Al or Cu foils using a green micro-processing kit (LVE-G10, Spectronix, Japan). A drop of ethylene glycol was spread over each Ag/Ti/Si substrate before mounting the dummy chip or mask to maintain the contact between the dummy chip or mask and the substrate. The specimens were then annealed at 250 °C for 1 h in a specific atmosphere, either the ambient atmosphere or an ultra-high vacuum (the pressure was lower than 10^−2^ Pa), without external pressure.

### Experimental Characterizations

After heat treatment at 250 °C for 1 h, microstructures of the samples were characterized utilizing optical microscopy, scanning electron microscopy, and transmission microscopy. Top views of abundant hillocks and pattern hillocks arrays were examined using an optical microscope (DM2700-M, Leica, Germany) and a field emission-scanning electron microscope (SU8020, Hitachi, Japan). Cross-sectional microstructures of abundant hillocks and Ag/Ag joints were revealed using conventional metallographic grinding and polishing processes and examined using a transmission electron microscope (JEM-2100, JOEL, Japan).

### Calculation Details

CALPHAD-type thermodynamic modeling was performed using the ThermoCalc Software. The thermodynamic description of the Ag-O binary system was taken from Assal *et al*.^10^ Four phases, including the liquid, face-centered cubic (fcc), Ag_2_O, and gas phases are considered. The liquid phase was modeled with an ionic liquid model with two sublattices, denoted as (Ag^1+^)_*p*_(O^2−^,Va^q−^)_*q*_, where *p* and *q* vary with composition, to maintain electroneutrality. The molar Gibbs energy of the liquid phase is described as


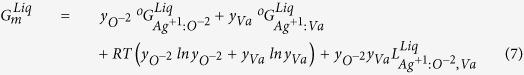


where *y*_*s*_ is the site fraction of s and 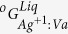 is the Gibbs free energy of pure liquid Ag, which is taken from the SGTE database compiled by Dinsdale^23^, 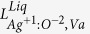
is the interaction parameter, and *R* and *T* are gas constant and temperature in Kelvin, respectively. The fcc phase was modeled with a substitutional solution model according to


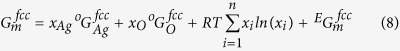


where 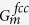 and *x*_*i*_ are the molar Gibbs free energy of the solution phase fcc and mole fraction of the species *i*, respectively. 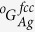 and 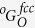 are the molar Gibbs free energy of the solution phase fcc composed of pure Ag and O, respectively, which are taken from the SGTE database^23^. 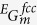
is the excess Gibbs free energy of the solution phase fcc and is described according to the Redlich-Kister polynomial. The Ag_2_O phase was modeled with a stoichiometric compound with two sublattices (Ag)_0.667_(O)_0.333_ according to


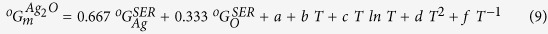


where 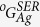 and 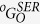 are the molar Gibbs free energy of pure Ag and O in their standard element reference (SER) states, respectively, and *a*, *b*, *c*, *d*, *f* are model parameters based on the formation enthalpy, entropy, and heat capacity in experiments. The gas mixture was modeled based on the SGTE database^23^. Equilibrium calculations were performed by including all the four phases, namely, the liquid, fcc-(Ag), Ag_2_O, and gas phases, while the metastable calculations were performed by suspending the Ag_2_O phase.

## Additional Information

**How to cite this article**: Lin, S.-k. *et al*. Nano-volcanic Eruption of Silver. *Sci. Rep*. **6**, 34769; doi: 10.1038/srep34769 (2016).

## Supplementary Material

Supplementary Information
