# Supplementary material for: Nano-volcanic Eruption of Silver
Source: Sci Rep. 2016 Oct 5;6:34769. doi: 10.1038/srep34769 (PMC5050507; doi:10.1038/srep34769)
Supplement: Supplementary Information [file srep34769-s1.pdf]

Electronic Supplementary Information for

Nano-volcanic Eruption of Silver

Shih-kang Lin,* Shijo Nagao,* Emi Yokoi, Chulmin Oh, Hao Zhang, Yu-chen Liu, Shih-guei Lin, Katsuaki Suganuma

*To whom correspondence should be addressed. E-mail: linsk@mail.ncku.edu.tw (S.K.L.); shijo.nagao@sanken.osaka-u.ac.jp (S.N.)

This PDF file includes:

Figs. S1 to S4

References

1. Formation of abundant Ag hillocks and roughened surfaces

Figure S1 (A) shows the optical micrograph of the edge of the “covered” region of a sputtered Ag film after being annealed at 250 °C for 1 h in the ambient atmosphere, and the close-ups of scanning electron micrographs of the “covered” (right) and “uncovered” (left) regions. A clear boundary between the “covered” and “uncovered” regions can be seen with an optical microscope. The different optical properties in the “covered” and “uncovered” regions are attributed from the surface microstructures, that abundant Ag hillocks form in the “covered” region (right), which roughens the surface and induces light scattering. Therefore, although the volume of abundant Ag hillocks are typically 1 to 2 μm^3, which has been beyond the spatial resolution of visible optics, the formation of abundant Ag hillocks can be clearly identified using a common optical microscope owing to the light scattering. Figure S1 (C) shows the schematic diagram of using a mask to produce patterned abundant Ag hillocks. The characters of masks are etched out using a laser micro-processing kit; hence, the characters shown in Figs. S1 (B) and (D) are the “uncovered” regions. The patterned abundant Ag hillocks in Figs. S1 (B) and (D) are

fabricated in the ambient atmosphere and in an ultra-high vacuum, respectively. For the former, more hillocks are found in the “covered” regions (inverse parts of characters), as shown in the close-up optical images in Fig. S1 (B). However, no hillocks can be found in the “covered” regions when annealing under an ultra-high vacuum, while uniformly formed hillocks can be found in the “uncovered” regions as shown in Fig. S1 (D). As the “Ag nano-volcanic eruption” mechanism being elucidated in the paper, it is not surprising to see this trend. It is also comprehensible that a sharper boundary between “covered” and “uncovered” regions is expected for the process under an ultra-high vacuum; that is, the thermal convection for the process in the ambient atmosphere may not cause blowing out all suspended Ag and Ag_2O clusters, so some of them still deposit at the “uncovered” region, resulting in the formation of a thinner amorphous Ag coating and subsequently fewer and smaller hillocks as shown in Fig. S1 (B). On the contrary, the autogenic mixture of suspended Ag and Ag_2O clusters and O_2 gas may be sucked into “uncovered” regions quickly, so no amorphous Ag coating nor hillocks will form,

which leaves a relatively flat surface behind at the “uncovered” regions (characters) as shown in Fig. S1 (D).

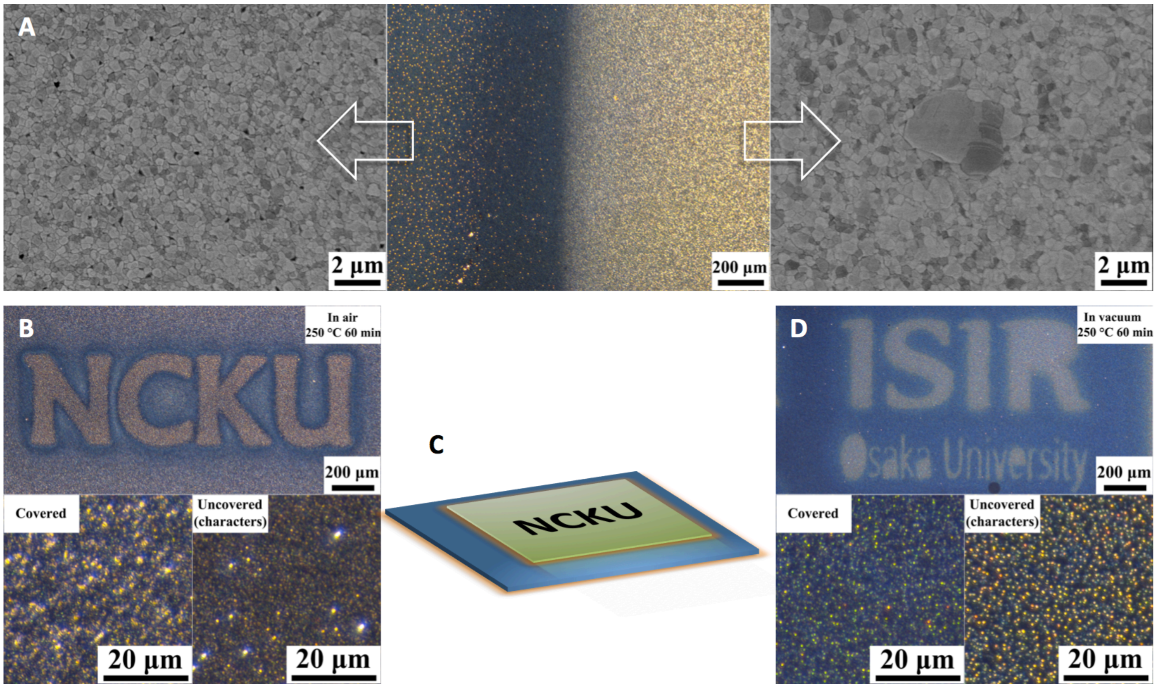

Fig. S1: (A) The optical micrograph of the edge of the “covered” region of a sputtered Ag film, which is annealed at 250 °C for 1 h in the ambient atmosphere, and the close-ups of scanning electron micrographs of the “covered” (right) and “uncovered” (left) regions. The mask was a dummy Si chip. (B) Optical micrographs of patterned abundant Ag hillocks, which were fabricated with a mask at 250 °C for 1 h in the ambient atmosphere, and its close-ups in “covered” and “uncovered” regions. (C) A schematic diagram of using a mask to produce patterned abundant Ag hillocks. (D) Optical micrographs of patterned abundant Ag hillocks, which were fabricated with a mask at 250 °C for 1 h in an ultra-high vacuum, and its close-ups in “covered” and “uncovered” regions.

2. Kinetic analyses of abundant Ag hillock formation

Adatom diffusion on free surface is much faster than both grain boundary diffusion and lattice diffusion. We can assume that all Ag atoms that arrive at the free surface would immediately join the hillock growth, as comparing to the mass transfers of Ag from the stressed Ag film to its free surface. The atomic flux (J) driven by stress gradient can be expressed as

$$J = \frac{D \cdot \Delta\sigma}{k \cdot T \cdot d} \quad (1)$$

where $\Delta\sigma$ is the stress difference and d is the film thickness, so $\Delta\sigma/d$ is the linear stress gradient, D is the diffusivity, T is the temperature, and k is the Boltzmann's constant.[24] The number of atoms (N') transported by the flux in a period of time t and through an area A is

$$N' = A \int_0^t J(t) dt \quad (2)$$

or the volume accumulated (V') through the stress-migration is

$$V' = \Omega \cdot A \int_0^t J(t) dt \quad (3)$$

where Ω is the atomic volume. Therefore, the volume accumulated (V') through the stress-migration is

$$V' = \frac{\Omega \cdot A \cdot D}{k \cdot T \cdot d} \int_0^t \Delta\sigma(t) dt \quad (4)$$

Since the atomic flux is contributed by both grain boundary diffusion and lattice diffusion, the accumulated volume through stress-migration can be further expressed as

$$V' = \frac{\Omega}{k \cdot T \cdot d} \int_0^t \Delta\sigma(t) dt \cdot \left[A_l \cdot D_l + A_{gb} \cdot D_{gb}\right] \quad (5)$$

where the subscript l and gb stand for lattice and grain boundary, respectively. For 1 μm-thick sputtered Ag films annealed at 250 °C for an hour, the $\int_0^t \Delta\sigma(t) dt$ can be estimated to be 8.26×10^{10} Pa · s.[12] Additionally, as depicted in Fig. S2, based on the density of hillock nucleation sites ($\sim 1.06 \times 10^5$ #/mm²) and average grain size of the Ag film (~104 nm) in experiments,[12] and the assumption of width of grain boundary to be 0.5 nm, the average areas of diffusion through lattice (A_l) and grain boundary (A_{gb}) per hillock can be estimated to be 9.33×10^{-12} m² and 9.38×10^{-14} m², respectively. By taking Ω, k, T, d, D_l, and D_{gb} to be 1.71×10^{-29} m³, 1.38×10^{-23} J/K, 523.15 K, 10^{-6} m, 5.61×10^{-24} m²/s, and 1.08×10^{-14} m²/s, respectively,[25,26] the estimated volume of each Ag hillock is ~0.2 μm³, which is significantly smaller than the actual abundant Ag hillocks (1~2 μm³) being observed in

experiments.[3,6,12,13]

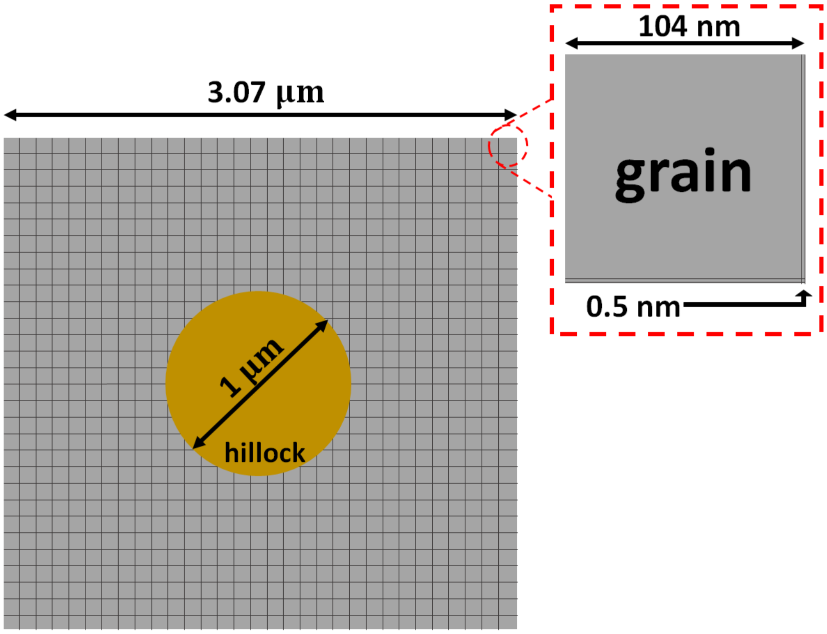

Fig. S2: The schematic top-view of the average territory a hillock on the Ag film with columnar grains.

3. *Ab initio* lattice stability of fcc-Ag and Ag_2O under external pressure

The Vienna *Ab-initio* Simulation Package (VASP)[27] using the density functional theory (DFT) with a plane wave basis was employed to simulate the lattice stability of fcc-Ag and Ag_2O phases under compressive stresses. Generalized gradient approximation (GGA) exchange-correlation functional and projector augmented wave (PAW) method[28] with an energy cut-off of 375 eV were used. The 11 x 11 x 11 k-point meshes and 2 x 2 x 2 *k*-point meshes for Brillouin zone sampling using Monkhost-Pack scheme[29] were performed for the 32-atoms Ag supercell[30] and 48-atoms Ag_2O supercell,[31] respectively. The numerical integration of the Brillouin zone and the energy cut-off were verified to produce absolute energy convergence to better than 10^{-3} eV/atom, with the forces at each atomic site converged to within 10^{-2} eV/Å. The dependence of strain energy on external pressure for each phase was calculated based on the total energy difference between the cell stressed structure and the fully relaxed structure. As shown in Fig. S3, the changes of lattice stability for both fcc-Ag and Ag_2O phases are only 0.4 and 0.3 meV/atom, respectively, when the external pressure is as large as 350 MPa, which was much

larger than that applied in any of our experiments. Therefore, as expected for most condensed phases, the changes in phase stability for both Ag and Ag_2O phases are negligible under very large stresses.

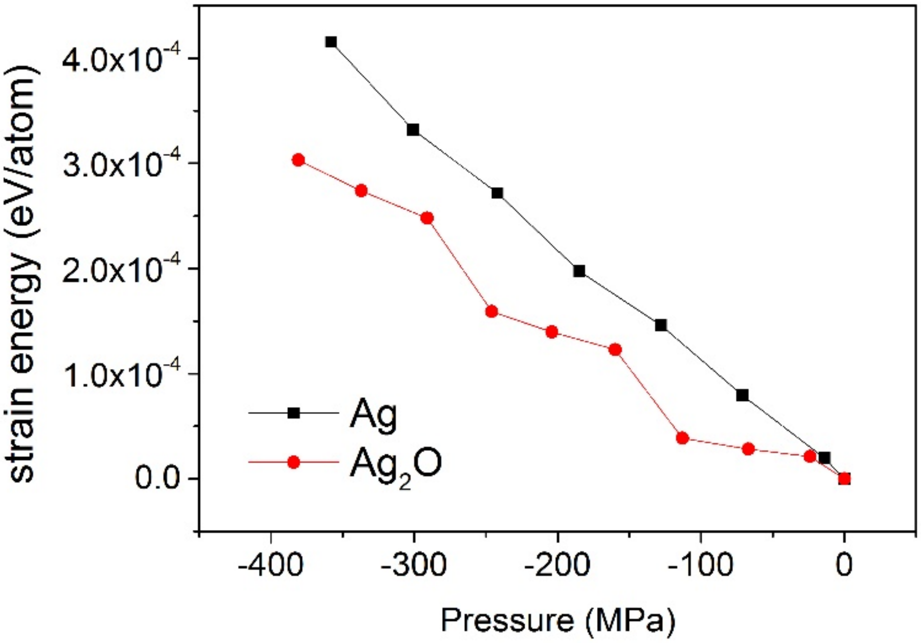

Figure S3: *Ab initio* strain energies of fcc-Ag and Ag_2O phases as function of external pressure ranging from 0 to 400 MPa.

4. Critical temperature on Ag nano-volcanic eruption

Ag-O grain boundary liquation due to the extremely high oxygen partial pressure at the grain boundaries is the first step of the “nano-volcanic eruption”. Figure S4 shows the optical micrographs of the sputtered Ag films, which are partially covered with dummy chips and annealed at 100, 120, 130, 140, 145, and 150 °C, respectively, in the ambient pressure for 5 h. After the prolonged annealing time, a clear boundary between the “covered” (right) and “uncovered” (left) regions can only be found in the sample, which was annealed at 150 °C. Evidently there is no nano-volcanic eruption and hillock formation after prolonged annealing at temperatures at or lower than 145 °C and vice versa. This critical temperature of approximately 150 °C is close to the normal decomposition temperature of Ag_2O. This finding strongly supports the “nano volcanic eruption” mechanism, as other theories concerning stress migration does not involve a critical temperature for formation of abundant Ag hillocks. Based on this understanding, an even lower processing temperature than 250 °C at as low as 150 °C is theoretically achievable for forming amorphous Ag coating as well as Ag-to-Ag direct bonding.

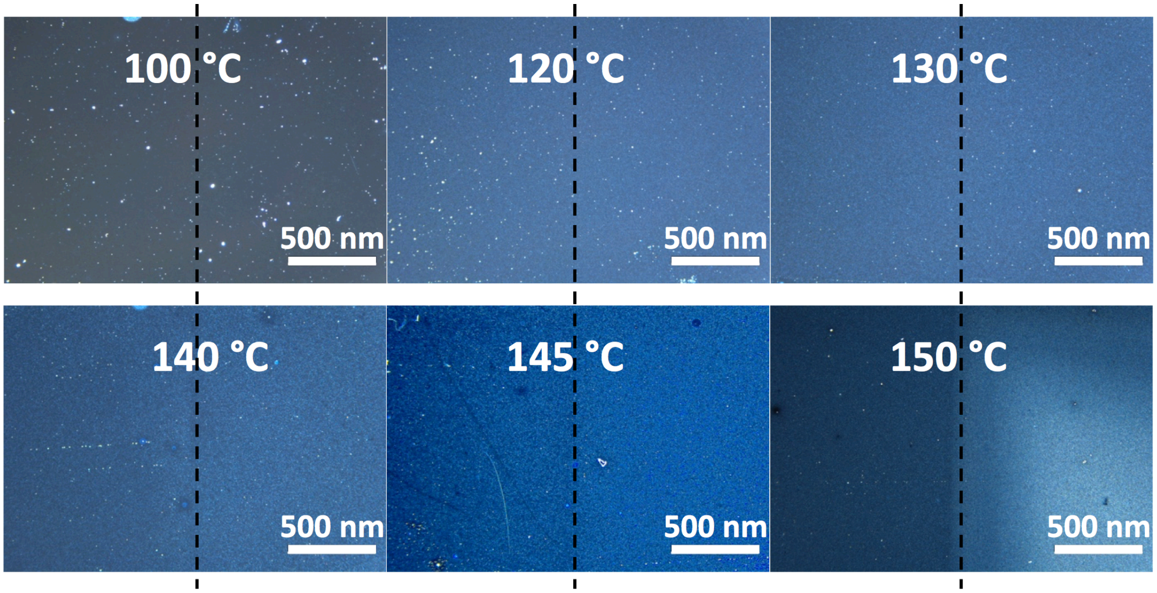

Figure S4: Optical micrographs of the sputtered Ag films, which are partially covered with dummy chips and annealed at 100, 120, 130, 140, 145, and 150 °C, respectively, in the ambient pressure for 5 h. The dashed lines indicate the edge of regions where were covered by dummy chips during heat treatment. A clear boundary can be seen in the sample annealed at 150 °C, while there are no noticeable differences in brightness for samples annealed at temperatures at or lower than 145 °C.

References

1. Ellingham, H. Transactions and Communications. *J. Soc. Chem. Ind.-L.* **63**, 125-160 (1944).
2. Sun, J. *et al*. Liquid-like Pseudoelasticity of Sub-10-nm Crystalline Silver Particles. *Nat. Mater.* **13**, 1007-1012, DOI:10.1038/nmat4105 (2014).
3. Oh, C., Nagao, S., Sugahara, T. & Suganuma, K. Hillock Growth Dynamics for Ag Stress Migration Bonding. *Mater. Lett.* **137**, 170-173, DOI:10.1016/j.matlet.2014.09.006 (2014).
4. Pi, S., Ghadiri-Sadrabadi, M., Bardin, J. C. & Xia, Q. Nanoscale Memristive Radiofrequency Switches. *Nat. Commun.* **6**, 7519, DOI:10.1038/ncomms8519 (2015).
5. Peng, P. *et al*. Joining of Silver Nanomaterials at Low Temperatures: Processes, Properties, and Applications. *ACS Appl. Mater. Inter.* **7**, 12597-12618, DOI:10.1021/acsami.5b02134 (2015).
6. Oh, C., Nagao, S., Kunimune, T. & Suganuma, K. Pressureless Wafer Bonding by Turning Hillocks into Abnormal Grain Growths in Ag Films. *Appl. Phys. Lett.* **104**, 161603, DOI:10.1063/1.4872320 (2014).
7. Assal, J., Hallstedt, B. & Gauckler, L. J. Thermodynamic Assessment of the Silver-oxygen System. *J. Am. Ceram. Soc.* **80**, 3054-3060 (1997).
8. Dinsdale, A. T. SGTE Data for Pure Elements. *Calphad* **15**, 317-425, DOI:10.1016/0364-5916(91)90030-n (1991).
9. Sun, Z., Hashimoto, H. & Barsoum, M. W. On the Effect of Environment on Spontaneous Growth of Lead Whiskers from Commercial Brasses at Room Temperature. *Acta Mater.* **55**, 3387-3396, DOI:10.1016/j.actamat.2007.01.035 (2007).
10. Ercker, L. *Treaties on Ores and Assaying (1574, 2nd ed. 1580), translated by Sisco AG, Smith CS. University of Chicago,* (1951).
11. Tu, K. N. Irreversible-process of Spontaneous Whisker Growth in Bimetallic Cu-Sn Thin-film Reactions. *Phys. Rev. B* **49**, 2030-2034, DOI:10.1103/PhysRevB.49.2030 (1994).
12. Oh, C., Nagao, S. & Suganuma, K. Pressureless Bonding Using Sputtered Ag Thin Films. *J. Electron. Mater.* **43**, 4406-4412, DOI:10.1007/s11664-014-3355-3 (2014).

13. Oh, C., Nagao, S. & Suganuma, K. Silver Stress Migration Bonding Driven by Thermomechanical Stress with Various Substrates. *J. Mater. Sci.-Mater. El.* **26**, 2525-2530, DOI:10.1007/s10854-015-2717-9 (2015).
14. Liu, C.-M. *et al*. Low-temperature Direct Copper-to-copper Bonding Enabled by Creep on Highly (111)-oriented Cu Surfaces. *Scripta Mater.* **78-79**, 65-68, DOI:10.1016/j.scriptamat.2014.01.040 (2014).
15. Liu, C.-M. *et al*. Low-temperature Direct Copper-to-copper Bonding Enabled by Creep on (111) Surfaces of Nanotwinned Cu. *Sci. Rep.* **5**, 9734, DOI:10.1038/srep09734 (2015).
16. Mimatsu, H. *et al*. Low-temperature Au-Au Bonding Using Nanoporous Au-Ag Sheets. *Jpn. J. Appl. Phys.* **52**, 1-4, DOI:10.7567/jjap.52.050204 (2013).
17. Lin, S.-K. *et al*. Mechanical Deformation-induced Sn Whiskers Growth on Electroplated Films in the Advanced Flexible Electronic Packaging. *J. Mater. Res.* **22**, 1975-1986, DOI:10.1557/jmr.2007.0232 (2007).
18. Lin, S.-K. *et al*. Microstructure Development of Mechanical-deformation-induced Sn Whiskers. *J. Electron. Mater.* **36**, 1732-1734, DOI:10.1007/s11664-007-0284-4 (2007).
19. Allen, N. P. The Effect of Pressure on the Liberation of Gases from Metals (with Special Reference to Silver and Oxygen). *J. I. Met.* **49**, 317-346 (1932).
20. Baker, E. H. & Johnston, J. K. Effect of Oxygen Pressure on Melting of Silver. *Nature* **205**, 65-66, DOI:10.1038/205065a0 (1965).
21. Johnston, J. K. Cryoscopic Studies of Melting Point of Silver in High Pressure Oxygen. *J. Electrochem. Soc.* **112**, C25, DOI:10.1149/1.2423510 (1965).
22. Baker, E. H. & Talukdar, M. I. Silver-oxygen System in the Pressure Range 0.2-750 atm, and Some Measurements on Silver-nitrogen and Gold-oxygen Equilibria. *T. I. Min. Metall.* **77**, C128-C133 (1968).
23. Michaelides, A., Bocquet, M. L., Sautet, P., Alavi, A. & King, D. A. Structures and Thermodynamic Phase Transitions for Oxygen and Silver Oxide Phases on Ag {111}. *Chem. Phys. Lett.* **367**, 344-350, DOI:10.1016/s0009-2614(02)01699-8 (2003).
24. Tu, K.-N. in *Electronic Thin-Film Reliability*. (Cambridge University Press), chap. 14, pp. 309-335 (2011).

25. Hoffman, R. E. & Turnbull, D. Lattice and Grain Boundary Self-diffusion in Silver. *J. Appl. Phys.* **22**, 634-639, DOI:10.1063/1.1700021 (1951).
26. Hoffman, R. E. & Turnbull, D. Erratum: Lattice and Grain Boundary Self-diffusion in Silver. *J. Appl. Phys.* **22**, 984-984, DOI:10.1063/1.1700085 (1951).
27. Kresse, G. & Furthmüller, J. Efficient Iterative Schemes for *Ab Initio* Total-energy Calculations Using a Plane-wave Basis Set. *Phys. Rev. B* **54**, 11169-11186 (1996).
28. Blöchl, P. E. Projector Augmented-wave Method. *Phys. Rev. B* **50**, 17953-17979 (1994).
29. Monkhorst, H. J. & Pack, J. D. Special Points for Brillouin-zone Integrations. *Phys. Rev. B* **13**, 5188-5192 (1976).
30. van Ingen, R. P., Fastenau, R. H. J. & Mittemeijer, E. J. Laser Ablation Deposition of Cu-Ni and Ag-Ni films: Nonconservation of Alloy Composition and Film Microstructure. *J. Appl. Phys.* **76**, 1871-1883, DOI:10.1063/1.357711 (1994).
31. Werner, A. & Hochheimer, H. D. High-pressure X-ray Study of Cu_2O and Ag_2O. *Phys. Rev. B* **25**, 5929-5934 (1982).
